# Kagome Quantum Oscillations in Graphene Superlattices

**DOI:** 10.1021/acs.nanolett.3c03524

**Published:** 2024-01-05

**Authors:** Folkert K. de Vries, Sergey Slizovskiy, Petar Tomić, Roshan Krishna Kumar, Aitor Garcia-Ruiz, Giulia Zheng, Elías Portolés, Leonid A. Ponomarenko, Andre K. Geim, Kenji Watanabe, Takashi Taniguchi, Vladimir Fal’ko, Klaus Ensslin, Thomas Ihn, Peter Rickhaus

**Affiliations:** 8 Laboratory for Solid State Physics, ETH Zürich, Zürich CH-8093, Switzerland; ‡ National Graphene Institute, University of Manchester, Manchester M13 9PL, United Kingdom; § Department of Physics & Astronomy, University of Manchester, Manchester M13 9PL, United Kingdom; ∥ ICFO-Institut de Ciencies Fotoniques, The Barcelona Institute of Science and Technology, Barcelona 08028, Spain; ⊥ Department of Physics, University of Lancaster, Lancaster LA1 4YW, United Kingdom; # National Institute for Materials Science, 1-1 Namiki, Tsukuba 305-0044, Japan; 7 Henry Royce Institute for Advanced Materials, Manchester M13 9PL, United Kingdom

**Keywords:** quantum oscillations, magneto-transport, moiré superlattice, graphene, electronic band structure

## Abstract

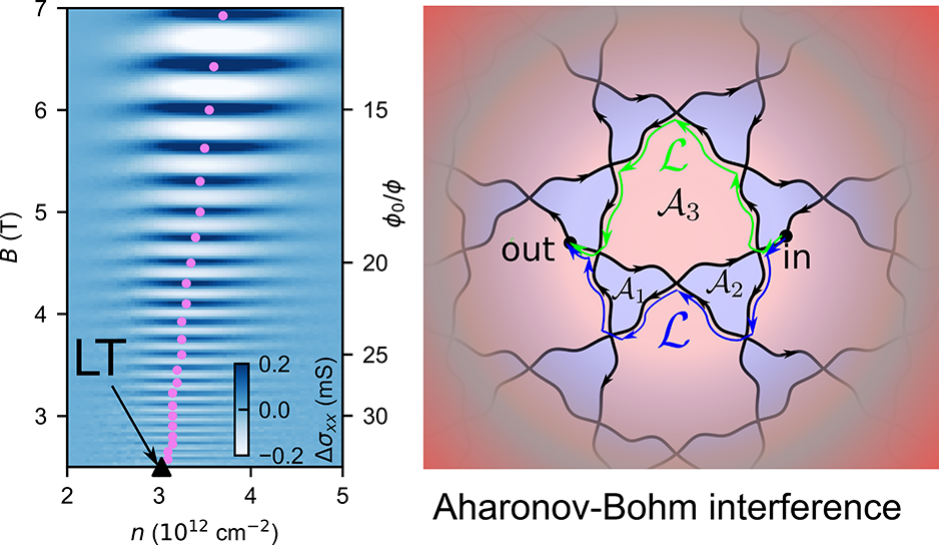

Electronic spectra of solids subjected to a magnetic field are often discussed in terms of Landau levels and Hofstadter-butterfly-style Brown–Zak minibands manifested by magneto-oscillations in two-dimensional electron systems. Here, we present the semiclassical precursors of these quantum magneto-oscillations which appear in graphene superlattices at low magnetic field near the Lifshitz transitions and persist at elevated temperatures. These oscillations originate from Aharonov–Bohm interference of electron waves following open trajectories that belong to a kagome-shaped network of paths characteristic for Lifshitz transitions in the moire superlattice minibands of twistronic graphenes.

Lifshitz transitions^1^ (LTs) are generic for electronic bands in solids. They mark the sign change of the effective band mass from “electron-like” to “hole-like”, accompanied by saddle-point dispersion features and van Hove singularities in the density of states. For two-dimensional (2D) crystals, a LT also singles out a band energy, ϵ(**p**) = *E*_LT_, for which disconnected closed-loop contours merge into a multiply-connected network extended over the entire reciprocal space. Figure 1a illustrates a characteristic LT contour for lattices that possess a C_3_ rotational symmetry. Its distinct trihexagonal kagome network form is generic for minibands formed by moiré superlattices (mSLs) in graphene-hBN heterostructures (G/hBN),^2−5^ twisted graphene bilayers,^6,7^ trilayers,^8−12^ and double-bilayers^13−16^ (tDBLG). It has a hexagonal part centered at γ and two triangular shapes around the κ and κ′ points of the mini Brillouin zone of the mSL. The corresponding hexagonal and triangular areas in Figure 1a are painted in red and blue, indicating which parts of a particular miniband dispersion are at the energies above and below *E*_LT_, respectively. The example shown in Figure 1 corresponds to the first mSL miniband on the conduction band side of the tDBLG spectrum. To represent the LT map for the first mSL miniband on the valence band side of tDBLG, one would have to swap red and blue colors; more examples are offered in Supporting Information (SI).

**Figure 1:**
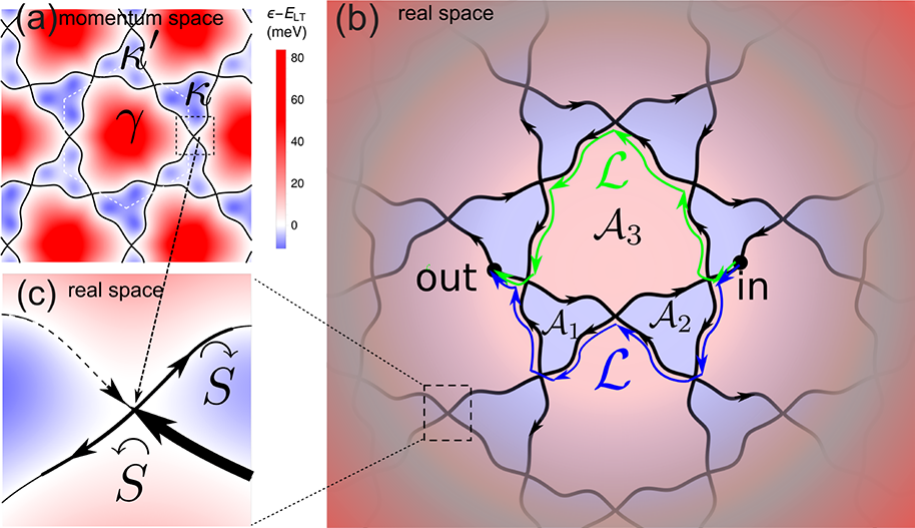
Characteristic reciprocal space (a) LT contour, ϵ = *E*_LT_, for the first miniband in a tDBLG, with (b) the corresponding trihexagonal kagome network of real-space ballistic trajectories of electrons in a magnetic field, fixed by the initial condition ”in” of the considered wave packet. Red and blue indicate miniband energies around κ, κ′, and γ above and below *E*_LT_ (in K′ valleys this map would be inverted). Green and blue lines in (b) exemplify the shortest paths (of the same length, ) responsible for the interference effect. (c) Bifurcations of real-space trajectories due to magnetic breakdown at the saddle points of the band dispersion.^17^

Constant energy maps are important for understanding magnetotransport phenomena, as they fully determine the shape of ballistic electron trajectories in a 2D metal subjected to a magnetic field, **B** = B**ẑ**. Because the electron’s dynamics is set by  and , the real-space trajectories can be obtained from constant-energy contours by a 90° rotation and rescaling using an (*eB*)^−1^ factor, Figure 1b. For closed-loop energy contours, this transformation results in cyclotron orbits, for which the Aharonov–Bohm interference leads to semiclassical Shubnikov–de Haas oscillations (SdHOs), the forerunners of Landau levels, and the quantum Hall effect. Here, we argue that multiply connected trajectories near LTs, formed through the magnetic breakdown effect,^17^ lead to a peculiar interference contribution to the conductivity which—unlike SdHOs—turns out to be insensitive to thermal broadening of the Fermi step, spanning over a finite interval of carrier densities around LTs. We refer to this behavior, here, observed experimentally in both tDBG and G/hBN structures, as “kagome oscillations” and identify those as the low-field forerunners of Brown–Zak minibands^18−20^ observed at high magnetic fields in quantum capacitance^3^ and magneto-transport.^4,5,21−23^

Let us consider an electron following a kagome network at the LT (such as in Figure 1b), where the propagation direction along each segment is set by the direction of the out-of-plane magnetic field, thus making the network chiral. Due to the quantum nature of electrons, their propagation along such a network mimics an interferometer. The meandering-like paths bifurcate at their intersections, Figure 1c, associated with the saddle-point features in the band. This branching is captured by transmission amplitudes:^25−30^ toward encircling κ or κ′ pockets and  toward the encircling γ pocket of the miniband dispersion.
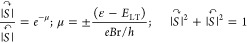
1where μ is the electron miniband energy counted from the LT toward the closest γ miniband edge (that is, we use “+” when κ and κ′ pockets are electron-like, as at the conduction band LT in tDBLG, and “–” when κ and κ′ pockets are hole-like) and normalized by a “magnetic breakdown” energy window, , set by the Gaussian curvature of the saddle-point dispersion, . Also, (arg S , where Γ is the Gamma-function.

Interference of electron waves, branched into, e.g., green and blue paths (with lengths ) in Figure 1b, affects the probability for an electron to reach an “out” state from an “in” state (see SI Section S4 for details), (The LT networks are not set in stone—they are linked to the initial position and velocity, |in⟩, of an electron, whereas the pairs of relevant (e.g., shortest) paths depend on the detection point |out⟩, as discussed in Figure S4SI Section S3
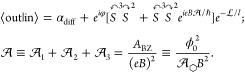
2These two paths are composed of equivalent ballistic segments explored in a different order (the powers of  and  are set by the numbers of left and right turns), making the electron-energy-dependent dynamical phase of the electron wave, , the same for both of them. This makes their mutual phase shift and the interference contribution set only by the encircled magnetic field flux,  (*A*_BZ_ and  are the mSL Brillouin zone area and unit cell area, respectively, and ϕ_0_ = *h*/*e*), independently of the electron’s energy deviation from *E*_LT_. This ballistic contribution comes on top of an amplitude, α_diff_, related to the diffusive propagation and a factor  that accounts for a suppression of ballistics by scattering by disorder and phonons. As , scattering is also the reason for considering the shortest paths in Figure 1b, in particular, at low magnetic fields. As a result, we arrive at an interference correction,
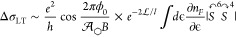
3to conductivity, σ_*xx*_ = σ_0_ + Δσ_LT_, which oscillates periodically as a function of *B*^–1^, spans from the LT point (e.g., as a function of carrier density), and sustains the thermal broadening, ∼*k*_*B*_*T*, of the Fermi step, *n*_*F*_(ϵ). The LT networks are not set in stone—they are linked to the initial position and velocity, |in⟩, of an electron, whereas the pairs of relevant (e.g., shortest) paths depend on the detection point |out⟩, as discussed in Figure S4 in SI. Note that for each pair of trajectories we account for the additional contribution of Maslov phases, as described in SI Section S3.

The features of the above-described interference contribution to conductivity can be recognized among the magneto-transport characteristics, Figure 2, observed in Hall-bar devices^24,32^ made of a 1.9°-twisted double-bilayer graphene (tDBLG). Figure 2a displays a color-scale map of low-temperature σ_*xx*_(*n*, *B*) with clear SdHOs fanned from both conduction and valence band edges of bilayer graphene and miniband edges (which are not reached within the explored range of carrier densities, *n*). The high conductivity on this map (at *B* = 0) points at the LT density in the sample, which agrees with the value estimated from the tDBLG dispersion^32^ (an icon in the panel) and coincides with the density where the low-field Hall conductivity (σ_*xy*_ in Figure 2b) changes sign.

**Figure 2:**
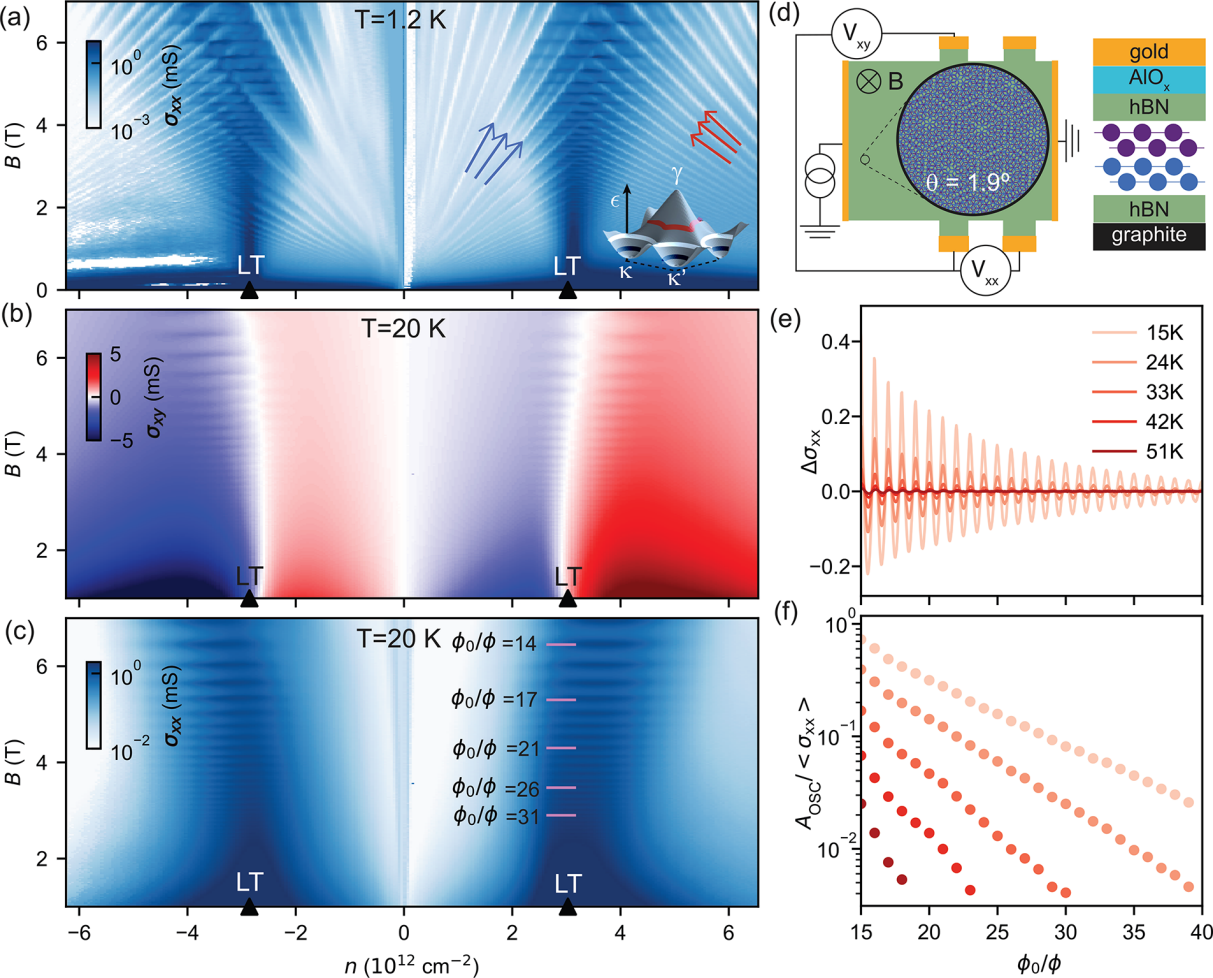
Magneto-transport oscillations in a double-gated tDBLG device sketched in (d). (a) Landau fan diagram σ_*xx*_(*n*, *B*) at *T* = 1.2 K. Arrows mark Landau levels formed around κ (blue) and γ (red) edges of the first miniband on the conduction band side shown in the inset. (b) σ_*xy*_ and (c) σ_*xx*_(*n*, *B*) measured at 20 K. Oscillations, periodic in ϕ_0_/ϕ, are pronounced in the vicinity of the LTs. (e) Oscillating part of conductivity, Δσ_*xx*_ at *n* = 3.2 × 10^12^ cm^–2^, and (f) the amplitude of these oscillations, plotted as a function of ϕ_0_/ϕ for various temperatures using color coding from (e) (see SI(24) for details).

In Figure 2c we show a typical conductivity map for the temperature range 10 K < *T* < 50 K where SdHOs (which reflect on the variation of the density of states of 2D electrons, ρ, due to the formation of Landau levels) are suppressed by thermal broadening of the Fermi step,  ∝  → 0,^35^ in particular, around LTs, where the density of states is high. The remaining high-temperature oscillations have σ_*xx*_ maxima when the magnetic flux through the mSL unit cell, , is commensurate, ϕ = ϕ_0_/*q* with an integer *q*, with the flux quantum, ϕ_0_ = *h*/*e*, independently of the gate-induced carrier density, *n*. The latter feature of the remaining oscillations formally coincides with the conditions for the formation of Brown–Zak minibands^18,19^ (also known as Hofstadter butterfly^20^). However, at the low magnetic field end and at elevated temperatures, the energy scale of the corresponding minibands and minigaps is much smaller than *k*_*B*_*T*. Hence, the observed effect does not reflect the density of states variation in the Brown–Zak minibands; instead, it points at an interference phenomenon present only in the electron transport characteristics. Also, these high-temperature oscillations in Figure 2c have the largest amplitude near the LTs, indicating that these are the above-described kagome oscillations.

To corroborate the generality of kagome oscillations in graphene superlattices, we compare, in Figure 3, oscillations observed around the LT in tDBLG with the earlier-observed high-temperature conductivity oscillations in a highly aligned graphene-hBN heterostructure (G/hBN),^4,5^ now traced to the low magnetic field range, 1 T < *B* < 3 T. In the latter system, the mSL has a 15 nm period and the LT in the first miniband at *n* ≈ −1.9 × 10^12^ cm^–2^ on the valence band side of graphene’s spectrum. In both data sets in Figure 3 one can notice that the kagome oscillations are visible over a finite interval of carrier densities near the LT which slightly shifts upon the increase of magnetic field. By inspecting Figure 2c, one can also notice that in tDBLG’s conduction and valence bands such a shift occurs in the opposite direction.

**Figure 3:**
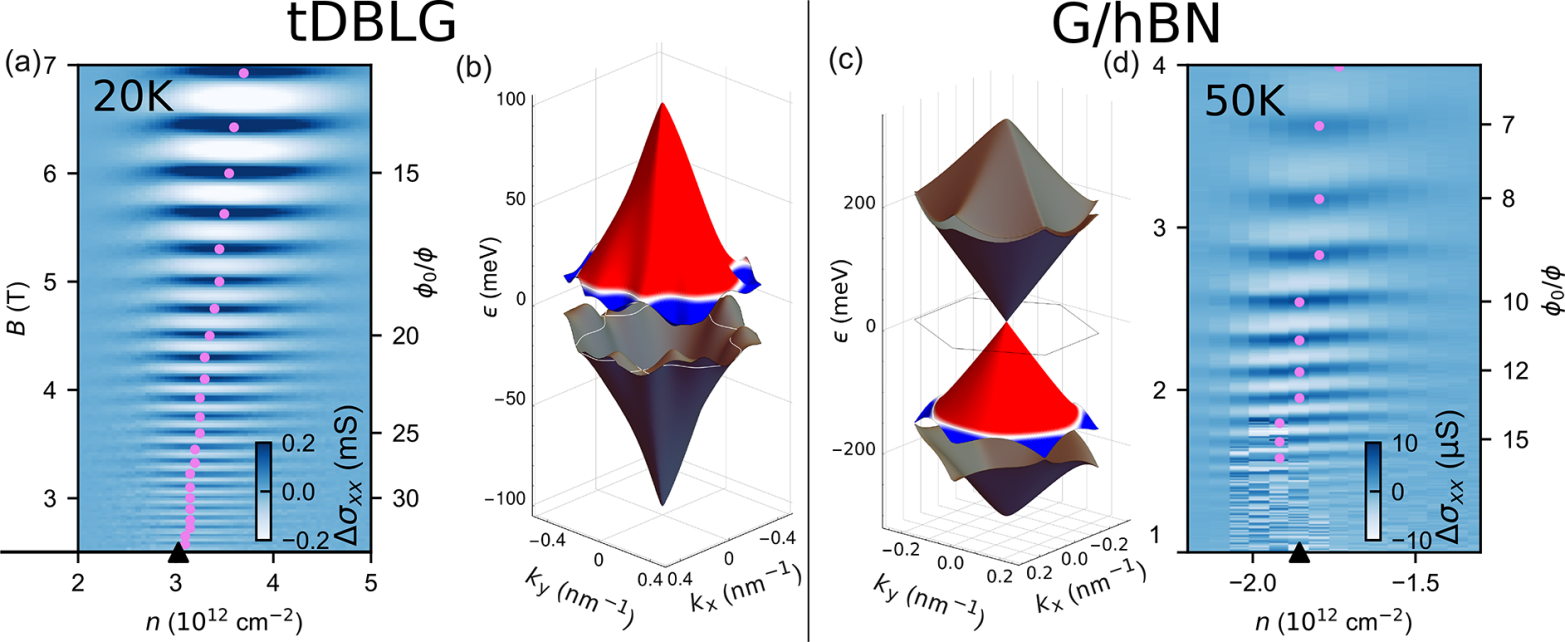
Similar behavior of σ_*xx*_ oscillations around LTs in moiré superlattice minibands in tDBLG and G/hBN. (a) Oscillations of σ_*xx*_( *n,B*) around the LT in the first miniband on the conduction band side of the tDBLG spectrum (b) computed using the Hamiltonian from refs (31 and 32) for a θ = 1.9° twist angle. The experimental data are plotted after subtracting a smooth background from data in Figure 2 (see ref (24) and Figures S2 and S3 for details); *T* = 20 K. (c) Electronic spectrum of G/hBN superlattices^33,34^) which features a clear LT in the first miniband on the valence band side of graphene’s spectrum (white contour). Dots mark the shift of the maxima of oscillations’ amplitudes upon the increase of the magnetic field.

The above-mentioned trend and its relation to common features of mSL minibands in C_3_-symmetric graphene superlattices can be understood using eqs 2 and 3. In those, an unbalanced product of right-turn and left-turn amplitudes reflects the trihexagonal shape of the LT networks, characteristic for the C_3_-symmetric band dispersions shown in Figure 3b,c (for other examples, see SI Figure S5).

The resulting factor, , in eq 3 has the energy dependence skewed toward the κ and κ′ pockets of the miniband dispersion. A saddle-point evaluation of the integral in eq 3, recalculated from the Fermi energy into carrier density dependence (using the density of states in the band, ρ, averaged over a *k*_*B*_*T* interval around the LT), results (see SI) in the following expression for the low-field oscillations,
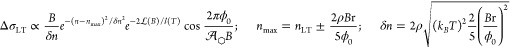
4

Here, ± accounts for the type of dispersion inside the trigonal pockets of the mSL Brillouin zone around κ and κ′: it is “+” for electron-like pockets, as shown in Figure 3b) and “–” for hole-like pockets, which is the case in the first miniband on the valence band side of tDBLG.

As a result, the inverted form of minibands on the valence and conduction band sides of the tDBLG spectrum sets the opposite shifts, , for the oscillations’ interval in n- and p-doped tDBLG (see Figure 3b), whereas the similar topology of the highlighted minibands in Figures 3b and 3c prescribes the same direction of a shift in the first miniband on the conduction band side of tDBLG (Figure 3a) and the first miniband on the valence band side of G/hBN (Figure 3d). Moreover, from the computed mSL minibands, we estimate  for tDBLG and for G/hBN^24^ and find that *δn* is temperature-limited up to *B* ∼ 10 T for tDBLG at *T* = 20 K and up to *B* ∼ 5 T for G/hBN at *T* = 50 K, explaining a weak *B*-dependence of the width of the regions where the oscillations occur in Figures 3a and 3d.

As the kagome oscillations come from the interference of ballistically propagating electrons, their amplitude is suppressed by scattering (both elastic and inelastic). In eq 3, this is accounted for by the factor , leading to an exponential decay of oscillations at low magnetic fields, which is a natural consequence of scaling, , of the lengths of kagome network segments. The exponential decay of the measured amplitudes of kagome oscillations, over 2 orders of magnitude, is apparent in Figures 2e and 2f (also see Figure S6 in SI), suggesting that those appear in the scattering-dominated transport regime. By fitting the measured magnetic field dependence of the oscillation amplitudes in Figure 2f with eq 4, we determine (*T*) for the studied temperature range, 10 K < *T* < 50 K, and find that it both is temperature-dependent—pointing toward the dominance of inelastic processes—and is shorter than the electron mean free path (estimated from conductivity at the LT and *B* = 0, see Figure S7 in SI); the latter feature is naturally expected, as  is affected by both large- and small-angle inelastic scattering via decoherence. Then, we use the obtained values of scattering length  to determine conditions for the formation of Brown–Zak minibands in the mSL, sketched in Figure 4. For Brown–Zak minibands to form, an electron needs to explore several magnetic supercells;^18,19,36^ hence, the scattering length  should be, at least, twice longer than the magnetic supercell perimeter,  (where *q* = ϕ_0_/ϕ), which is reflected by the dashed line on the *B*–*T* plane.

**Figure 4:**
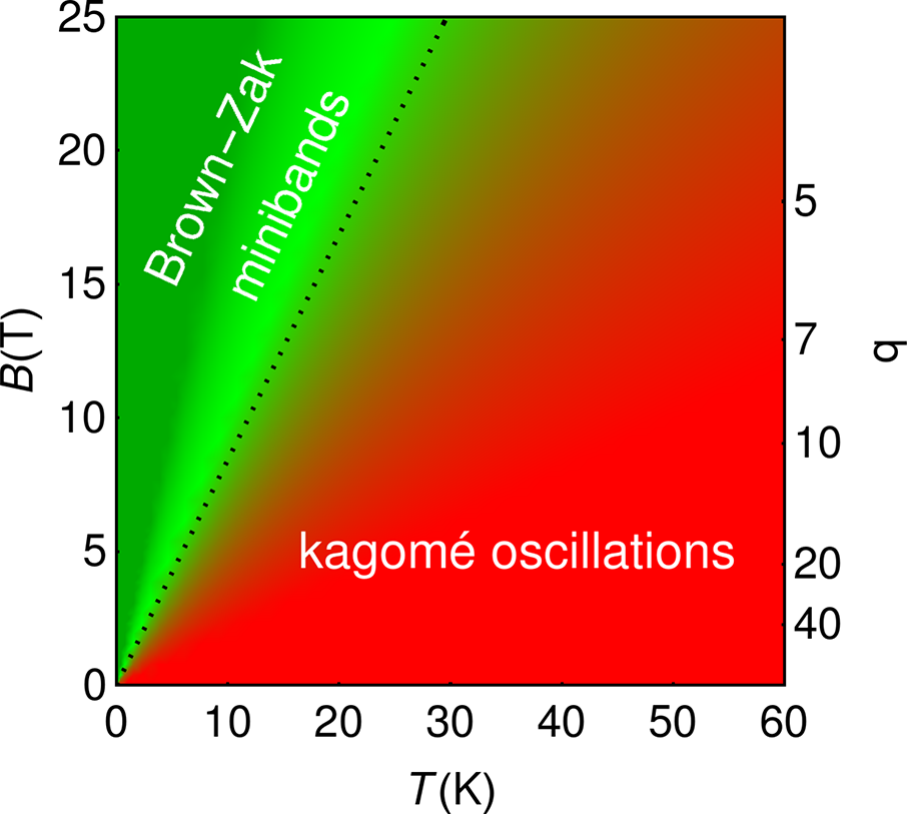
Crossover between kagome oscillations and Brown–Zak miniband regimes in the graphene mSL at *n* ≈ *n*_LT_. The dotted line corresponds to B* such that , with the values  obtained from fitting the data in Figure 2f using eq 4. For *B* < B*, Brown–Zak minibands are undermined by scattering, whereas kagome oscillations are detectable, though with an exponentially small amplitude; *B* > B* corresponds to the truly ballistic regime where Brown–Zak minibands are stabilized.

While the studies of kagome oscillations in this paper were focused on moiré superlattices with trigonal (C_3_) symmetry, we expect similar forerunners of Brown–Zak oscillations to exist in crystals with a C_4_ symmetry,^20,37^ too. However, we note that they would be suppressed in systems with lower symmetry. This is because LT contours in low-symmetry crystals have the form of quasi-1D block-chains of intertwining meanders^26,38^ which do not provide pairs of paths needed for the energy-independent Aharonov–Bohm interference. Therefore, breaking the C_3_ rotational symmetry of the mSL by straining one of the 2D crystals in a stack^39,40^ and violating the kagome topology of the LT network would suppress these novel low-magnetic-field high-temperature quantum oscillations.

## Supporting Information Available

Materials and Methods, including: tDBLG device characterization and experimental data analysis; G/hBN device characterization and data analysis; Maslov and Berry phases; shortest interfering paths; details of saddle-point calculation, leading to eq 4; calculation of dispersion for tDBLG and G/hBN; and temperature dependence of kagome oscillation amplitude and estimate of coherence length. Figure S1: σ_*xx*_(*n*, *B*) map at low temperature. Figure S2: Details of background subtraction procedure used to extract oscillations in the experiment. Figure S3: Detailed experimental data on doping dependence of oscillation amplitude. Figure S4: Illustration of shortest interfering paths. Figure S5: Fermi contours at the Lifshitz transition are compared between several different C_3_ symmetric systems. Figure S6: Full experimental data and a fit for oscillation amplitude at different temperatures and magnetic fields.
