# Supplementary material for: Kagome Quantum Oscillations in Graphene Superlattices
Source: Nano Lett. 2024 Jan 5;24(2):601–6. doi: 10.1021/acs.nanolett.3c03524 (PMC10797620; doi:10.1021/acs.nanolett.3c03524)
Supplement: Supplementary file 1 — nl3c03524_si_001.pdf [file nl3c03524_si_001.pdf]

Supplementary for "Kagomé quantum oscillations in graphene superlattices"

Folkert K. de Vries,[1,*] Sergey Slizovskiy,[2,3,†] Petar Tomić,[1] Roshan Krischna Kumar,[2,3,4] Aitor Garcia-Ruiz,[2,3] Giulia Zheng,[1] Elías Portolés,[1] Greg H. Auton,[2,3] Leonid A. Ponamorenko,[5] Andre K. Geim,[2,3] Kenji Watanabe,[6] Takashi Taniguchi,[7] Vladimir Fal'ko,[2,3,8] Klaus Ensslin,[1] Thomas Ihn,[1] and Peter Rickhaus[1]

[1] *Laboratory for Solid State Physics, ETH Zürich, CH-8093 Zürich, Switzerland*
[2] *National Graphene Institute, University of Manchester, Manchester M13 9PL, United Kingdom*
[3] *Department of Physics & Astronomy, University of Manchester, Manchester M13 9PL, United Kingdom*
[4] *ICFO-Institut de Ciencies Fotoniques, The Barcelona Institute of Science and Technology, Barcelona, Spain*
[5] *Department of Physics, University of Lancaster, Lancaster LA1 4YW, United Kingdom*
[6] *Research Center for Functional Materials, National Institute for Materials Science, 1-1 Namiki, Tsukuba 305-0044, Japan*
[7] *International Center for Materials Nanoarchitectonics, National Institute for Materials Science, 1-1 Namiki, Tsukuba 305-0044, Japan*
[8] *Henry Royce Institute for Advanced Materials, M13 9PL, Manchester, United Kingdom*

(Dated: December 17, 2023)

Supplementary Materials include: Materials and Methods:

- tDBLG device characterization and experimental data analysis
- G/hBN device characterization and data analysis
- Maslov and Berry phases
- Shortest interfering paths
- Details of saddle-point calculation, leading to Eq. (4)
- Calculation of dispersion for tDBLG and G/hBN
- Temperature dependence of kagome oscillation amplitude and estimate of coherence length

Figures:

- S1: $\sigma_{xx}(n, B)$ map at low temperature.
- S2: Details of background subtraction procedure used to extract oscillations in the experiment.
- S3: Detailed experimental data on doping dependence of oscillation amplitude.
- S4: Illustration of shortest interfering paths
- S5: Fermi contours at Lifshitz transition are compared between several different C_3 symmetric systems.
- S6: Full experimental data and a fit for oscillation amplitude at different temperatures and magnetic fields.

S1. TDBLG DEVICE CHARACTERIZATION AND EXPERIMENTAL DATA ANALYSIS

For this work we used the same device as reported on in Ref. [1]. Details on the fabrication process, as well as optical images of the Van der Waals stack can be found there. We measured $V_{\rm xx}$ and $V_{\rm xy}$, convert this to $\rho_{\rm xx}$ and $\rho_{\rm xy}$ using the applied current I and the width of the mesa $W = 2374\,\text{nm}$ and the average distance between the contacts of $L = 910\,\text{nm}$, and obtain $\sigma_{\rm xx}$ and $\sigma_{\rm xy}$ through tensor inversion. We control the temperature in a pumped Helium-4 cryostat by a heater and a feedback loop, allowing us to reach stable values from 1.2 K up to 60 K.

* devriesf@phys.ethz.ch
† Second author contributed equally
Sergey.Slizovskiy@manchester.ac.uk

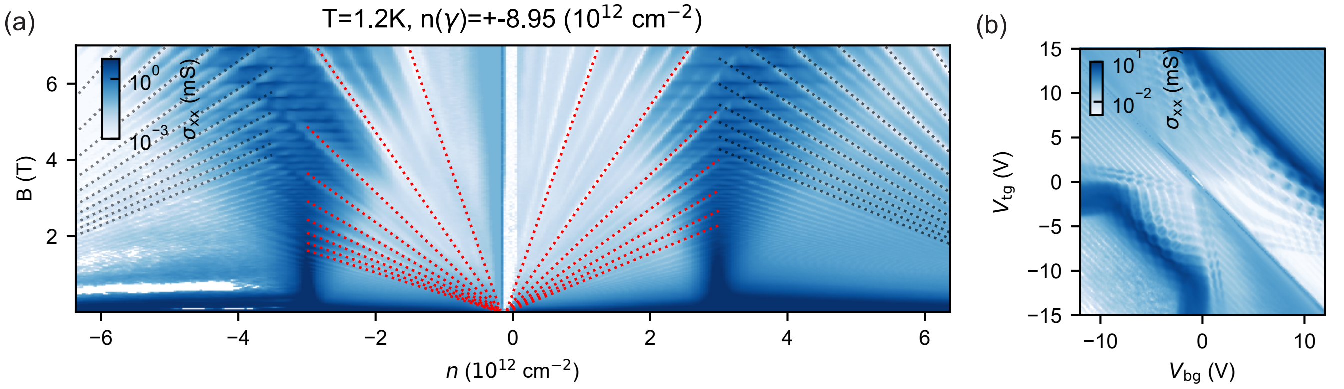

FIG. S1. (a) Shubnikov–de Haas map, $\sigma_{xx}(n, B)$ at zero displacement field, as plotted before in Fig.1(a). Here the SdH oscillations are fit (red and black dashed lines) to extract the degeneracies and full filling density $n(\gamma)$ as indicated. (b) σ_{xx} versus both bottom gate (V_{bg}) and top gate (V_{tg}) voltages at a constant magnetic field of $B = 2\,\mathrm{T}$. Shubnikov–de Haas oscillations indicate constant density lines for the two bilayers.

We calculate the density (in cm^{-2}) and displacement field (in V/nm) using the following equations:

$$n = \frac{1}{e}(C_{bg}V_{bg} + C_{tg}V_{tg}) \cdot 10^{-4} - n_{offset}, \tag{S1}$$

$$D = -\frac{0.5}{\epsilon_0}(C_{tg}V_{tg} - C_{bg}V_{bg}) \cdot 10^{-9} \tag{S2}$$

where C_{xx} are the capacitance to the respective gates, e is the elementary charge, ϵ_0 the vacuum permittivity, and n_{offset} an offset in the density. The capacitances are obtained in two steps. First we estimate the capacitance per area between the gate and the TDBG using the parallel plate capacitor model. Input parameters are the thickness of the hBN and AlO_x layers as well as their dielectric constants. Then we finetune the capacitance found by measuring the Landau levels as a function of the estimated density, and fitting the Landau levels using $n = h/q \cdot B/\nu$ where q is either +e for holes or -e for electrons (Fig. S1(a)). To crosscheck the ratio of the capacitances to top and bottom gate we then perform a measurements of the SdHO at constant magnetic field and as a function of both gates (Fig. S1(b)). The slope of the line where the total density equals zero gives us the ratio of the capacitances.

Furthermore, the fit of the Shubnikov–de Haas oscillations in Fig. S1 allows us to check the degeneracies. The red dashed lines correspond to SdHO with a degeneracy of 8. The degeneracy corresponds to spin, valley and minivalley (or layer) degree of freedom. The data reveals that this degeneracy is broken at higher magnetic fields where three additional lines appear between each pair of red dashed lines. This corresponds to the situation where the spin- and valley degeneracy is lifted, while the mini-valley degeneracy is maintained even at 7 T.

Finally, the oscillations emerging from large densities (i.e. $8.95 \times 10^{12}\,\mathrm{cm}^{-2}$) are four-fold degenerate, as indicated by the black dashed lines. The change from eight-fold degenerate electrons (at $n > 0$) to four-fold degenerate holes occurs at the Lifshitz transition. These fits allow us to estimate the density at full band filling and from that calculate a twist angle of 1.9°. We cross-checked this with the Brown–Zak oscillations and were able to refine it to 1.94°.

The background subtraction procedure used to plot Fig. 3 (a) in the main text is shown in Fig. S2. The kagom'e oscillations are visible in Fig. S2, where we show $\sigma_{xx}(n, q)$ at 20 K. For better visibility, we apply a background subtraction procedure. We use a smoothing filter (Savitzky-Golay) along the n-axis (27 points, 1st order) and obtain the background $\langle\sigma_{xx}\rangle$ plotted in Fig. S2(b). By subtracting the two maps from each other, we obtain Fig. S2(c), i.e. $\Delta\sigma_{xx} = \sigma_{xx} - \langle\sigma_{xx}\rangle$. The exact procedure is used for different temperatures for Fig. 3(e) and to obtain Fig. 3(d) smoothing is done over 19 points instead.

Next, we obtain the kagome oscillation amplitude $A_{osc}(n)$, used for Fig. 2(f). We first extract cuts $\sigma_{xx}(n)$ at integer q and half-integer q, see Fig. S3(a). Then from these, we calculate $A_{osc} = \sigma_{xx}(q) - {}^1\!/_2(\sigma_{xx}(q + {}^1\!/_2) + \sigma_{xx}(q - {}^1\!/_2))$, i.e. A_{AB} is given by constructive minus destructive interference, see Fig. S3(b). To obtain A_{AB} for many different temperatures for Fig. 2(f) we have taken linetraces at different temperatures at the density indicated in Fig. S3(b).

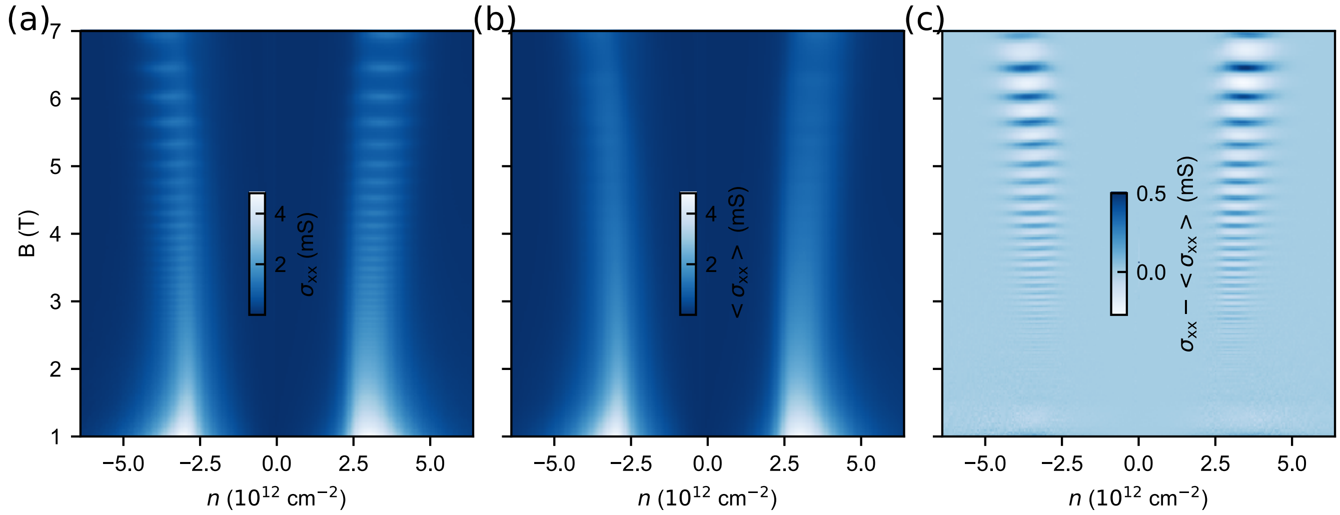

FIG. S2. Background subtraction used for Fig. 2(c). (a) obtained $\sigma_{\rm xx}(n,B)$, (b) smoothed background $\langle\sigma_{\rm xx}(n,B)\rangle$, (c) $\Delta\sigma_{\rm xx}(n,B)$, the difference between (a) and (b).

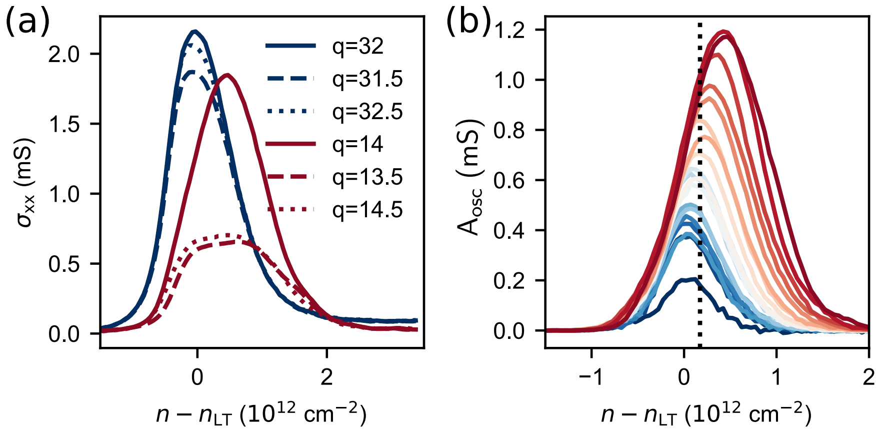

FIG. S3. (a) Examples of $\sigma_{\rm xx}$ traces at half integer and integer q. (b) The extracted amplitude of the oscillations $A_{\rm AB}$ for q ranging from 14 (red) to 32 (blue). The dashed line indicates the density at which the amplitude data for the temperature dependent measurements is taken. Both panels are for $T = 15\,{\rm K}$.

S2. G/HBN DEVICE CHARACTERIZATION AND DATA ANALYSIS

The data for graphene aligned on hBN at $T = 50$ K (Fig. 3d of main text) is a previously unpublished data, obtained in the experiment reported in Ref.[2]. The device details can be found in Supplementary Section 1 of Ref.[2]. The oscillatory part of σ_{xx} has been extracted by the same method as for tDBLG sample discussed above.

S3. MASLOV AND BERRY PHASES

The phases of the amplitudes $\overset{\frown}{S}$ and $\overset{\frown}{S}$, presented in Eq.(1), do not include the Maslov phase, which should be included in the amplitude as $-i$ multiplier for each clock-wise p_y turning point and as i for each anti-clock-wise p_y turning point [3]. Such separation of Maslov phases allows us to describe all the differently-oriented saddle points in a gauge-independent way.

A more precise version of semiclassical formalism includes also the Berry curvature and Berry magnetic moment corrections to the semiclassical phase, see [4–7]. Effectively, the Aharonov–Bohm interference correction that we describe corresponds to a phase gained on a contour encircling the Brillouin zone. The Berry magnetic moment can

be accounted for by a magnetic field dependent shift of the dispersion. The Berry phase, which is typically written in momentum space as $e^{i\oint \mathbf{A}_{Berry}(p)\cdot d\mathbf{p}} = e^{2\pi i N} = 1$, does not contribute because its integral around the Brillouin zone area equals an integer Chern number, N, of the band. Gauge-invariant Roth and Zeeman phases appearing in the semiclassical formalism [6, 8] would also not contribute, because for each traversed segment, the loop contains an equivalent segment (shifted by a reciprocal lattice vector) that is traversed in the opposite direction.

S4. SHORTEST INTERFERING PATHS

In this section, we describe all the shortest interference paths contributing to kagome oscillations. We start with a random phase space point "in", as shown in Fig.S4, and show all 4 of the possible shortest interfering contributions. For "out" points chosen on any other segments (not shown in Fig.S4), the Aharonov–Bohm interference contribution will contain longer path and will not be dominant in the limit of short phase coherence.

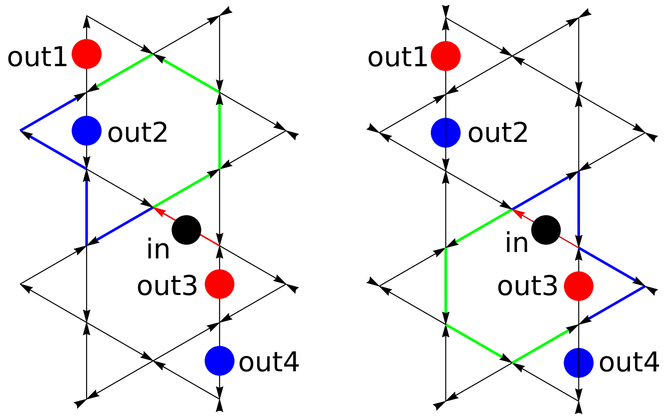

FIG. S4. Schematic illustration of 4 shortest-path amplitudes producing kagome oscillations in conductance. Black dot is a starting point on a randomly selected segment, red dots indicate final segments for which probabilities get a $+\cos(2\pi\phi_0/\phi)$ interference correction (due to the real Maslov phase) and blue dots correspond to $-\cos(2\pi\phi_0/\phi)$ terms (due to the imaginary Maslov phase). Interfering paths are shown in green and blue (for "out1" and "out2" on the left and for "out3" and "out4" on the right). Adding up the 4 leading contributions shows that "+" terms dominate in the conductance, leading to $+\cos(2\pi\phi_0/\phi)$ oscillations in the conductance.

Explicitly, the amplitudes are

$$\langle \text{out1}|\text{in}\rangle = \alpha_{\text{diff}} + e^{i\phi}\left[\overset{\curvearrowleft}{S}^3\overset{\curvearrowright}{S}^2 + \overset{\curvearrowleft}{S}^3\overset{\curvearrowright}{S}^2 e^{i\frac{eB(\mathcal{A}_1+\mathcal{A}_2+\mathcal{A}_3)}{\hbar}}\right] e^{-\frac{\mathcal{L}_{\text{in-out1}}}{2\ell}} \tag{S3}$$

$$\langle \text{out2}|\text{in}\rangle = \alpha_{\text{diff}} + e^{i\phi}\left[-i\overset{\curvearrowleft}{S}^2\overset{\curvearrowright}{S}^3 + i\overset{\curvearrowleft}{S}^4\overset{\curvearrowright}{S} e^{i\frac{eB(\mathcal{A}_1+\mathcal{A}_2+\mathcal{A}_3)}{\hbar}}\right] e^{-\frac{\mathcal{L}_{\text{in-out2}}}{2\ell}} \tag{S4}$$

$$\langle \text{out3}|\text{in}\rangle = \alpha_{\text{diff}} - e^{i\phi}\left[\overset{\curvearrowleft}{S}\overset{\curvearrowright}{S}^4 + \overset{\curvearrowleft}{S}^5 e^{i\frac{eB(\mathcal{A}_1+\mathcal{A}_2+\mathcal{A}_3)}{\hbar}}\right] e^{-\frac{\mathcal{L}_{\text{in-out3}}}{2\ell}} \tag{S5}$$

$$\langle \text{out4}|\text{in}\rangle = \alpha_{\text{diff}} + e^{i\phi}\left[-i\overset{\curvearrowleft}{S}^2\overset{\curvearrowright}{S}^3 + i\overset{\curvearrowleft}{S}^4\overset{\curvearrowright}{S} e^{i\frac{eB(\mathcal{A}_1+\mathcal{A}_2+\mathcal{A}_3)}{\hbar}}\right] e^{-\frac{\mathcal{L}_{\text{in-out4}}}{2\ell}} \tag{S6}$$

where we have taken into account the Maslov phases. When module-squared to get the probability, all the 4 amplitudes presented above produce an oscillatory term $\sim \pm\overset{\curvearrowleft}{S}^6\overset{\curvearrowright}{S}^4\cos(2\pi\phi/\phi_0)$ and the sign is $+$ for "out1" and "out4" and $-$ for "out2" and "out3".

There are two possible approaches to extract conductivity: first is the Einstein relation between conductivity and diffusion, which implies that a contribution to the conductivity is proportional to the squared distance, $\langle x^2\rangle$, covered between the two scatterings. Since the contributions with $+$ sign are seen to have higher values of $\langle x^2\rangle$, the overall sign of oscillating term is $+\cos(2\pi\phi_0/\phi)$. Another approach to conductivity is based on Kubo formula and involves the velocity-velocity correlator. Since the positive (negative) contributions have velocities with positive (negative) projection onto the initial velocity, the total contribution is, clearly, positive.

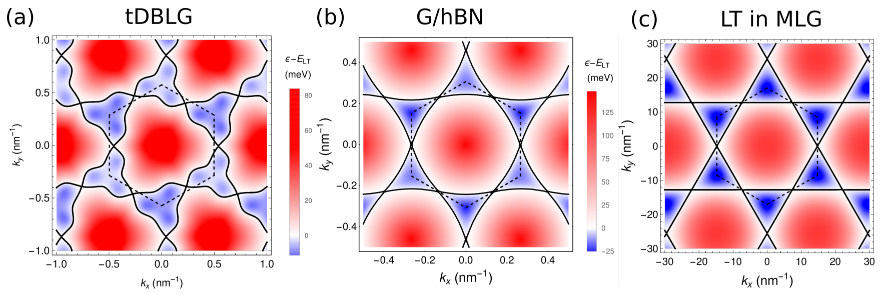

FIG. S5. Comparison of Fermi contours at LT between (a) 1.9° tDBLG (conductance band), (b) valence band of G/hBN, (c) graphene monolayer doped up to LT In all the cases we see the same LT topology where one FS turns into two complimentary ones.

S5. DETAILS OF SADDLE-POINT CALCULATION, LEADING TO EQ.(4)

The amplitude of kagome oscillations is clearly peaked near the LT since it involves a product of both $\overset{\curvearrowright}{S}$ and $\overset{\curvearrowleft}{S}$ terms, the typical energy width of oscillation region is $\sim \hbar e B \mathfrak{r}$. A distinguishing feature of hexagonal network is that the maximum amplitude of oscillations is shifted from the Lifshitz transition point ($\mu = 0$) in the direction of a higher doping (as shown in Fig. 2c and Fig. 3 a,d of the Main Text). This occurs because higher power of $\overset{\curvearrowleft}{S}$ compared to $\overset{\curvearrowright}{S}$ is involved in the oscillation amplitude, Eq.(2), $\left|\overset{\curvearrowleft}{S}{}^6 \overset{\curvearrowright}{S}{}^4\right| = \frac{e^{\mu/2}}{32\cosh(\mu/2)^5}$. The maximum of this expression is shifted by

$$\epsilon_{\max} = -\frac{eB\mathfrak{r}\ln(3/2)}{2\pi\hbar} = -\frac{\mathfrak{r}B}{\phi_0}\ln\frac{3}{2} \tag{S7}$$

from the energy of Lifshitz transition. The peak in $\left|\overset{\curvearrowleft}{S}{}^6 \overset{\curvearrowright}{S}{}^4\right|$ is further broadened by accounting for the finite temperature. To evaluate the integral analytically, we can expand the exponents in Taylor series either around the LT ($\epsilon = 0$), or around the maximum ($\epsilon_{\max}$), or, one can approximate the integrand with a Gaussian according to a mean value and dispersion. All these approaches lead to very similar results that differ numerically by a few percent. Taylor-expanding the exponent around the LT point and also approximating the n'_F with a Gaussian gives

$$\int d\epsilon\, n'_F(\epsilon)|\overset{\curvearrowleft}{S}{}^6 \overset{\curvearrowright}{S}{}^4| \propto$$
$$\int \frac{d\epsilon}{T}\,\exp\left[-\frac{5\pi^2\hbar^2}{2e^2\mathfrak{r}^2B^2}\left(\epsilon + \frac{2\mathfrak{r}B}{5\phi_0}\right)^2\right]\exp\left[\frac{-(\epsilon-\epsilon_F)^2}{4T^2}\right],$$

leading to Eq.(4) of the Main Text. Note that the resulting position of the maximum in Eq.(4), $\epsilon_{\max\ (\mathrm{saddle})} = -\frac{2}{5}\frac{\mathfrak{r}B}{\phi_0}$ is numerically very close to the value in Eq.(S7), because $\ln(3/2) = 0.4055 \approx 2/5$.

S6. CALCULATION OF DISPERSION FOR TDBLG AND G/HBN

Although our results do not depend on the details of dispersion, we used the dispersion relations to plot the network and estimate the Gaussian curvature near the saddle-points. The dispersion of tDBLG was calculated according to Refs.[9], and the details can be found in Supplementary to Ref. [1] (parameters of Slonczewski-Weiss-McClure model used are $\gamma_0 = 3.16\,\mathrm{eV}$, $\gamma_1 = 0.381\,\mathrm{eV}$, $\gamma_3 = -0.38\,\mathrm{eV}$, $\gamma_4 = 0.14\,\mathrm{eV}$). For aligned G/hBN system, we used Refs. [10] (parameter values $U_0 = 8.5\,\mathrm{meV}$, $U_1 = -17\,\mathrm{meV}$, $U_3 = -14.7\,\mathrm{meV}$).

The kagome networks of saddle-point trajectories in magnetic field look very similar for tGBLG and G/hBN and shown in Fig.S5.

S7. TEMPERATURE DEPENDENCE OF KAGOME OSCILLATION AMPLITUDE AND ESTIMATE OF COHERENCE LENGTH

In Fig. S6 we show the full dataset for oscillation amplitude at doping where it is maximal, $n \approx n_{max}$. The minimal real-space path required to see the oscillations can be estimated by taking the length of a green path in Fig.1

(same as the length of a blue path) in momentum space, and rescaling that into the real space length using a factor $1/(eB)$, resulting in $\mathcal{L} \approx \frac{\hbar}{eB}\, 2\,\mathrm{nm}^{-1}$. Note that $2\mathcal{L}$ is comparable to the perimeter of an extended magnetic supercell, $\mathcal{L}_q = 4\sqrt{\frac{2}{\sqrt{3}}} q \sqrt{\mathcal{A}_{\hexagon}} = \frac{\hbar}{eB}\, 3.98\,\mathrm{nm}^{-1}$, where $\mathcal{A}_{\hexagon} \approx 46\,\mathrm{nm}^2$ for the studied tDBLG.

Fitting the measured magnetic field dependence of oscillations amplitudes with Eq.(4) and length $\mathcal{L}$, we extract the value of the scattering parameter, $\ell^{-1}(T)$, which determines the loss of electrons from ballistic propagation. The result of such fitting are displayed as black circles in Fig.S7, where we also compare it with the inverse of the mean free path, ℓ^{-1}_{mfp} (blue curve), determined from the conductivity measured at the Lifshitz transition density and $B = 0$ (here, we use average velocity estimated from the computed dispersion, shown in Fig. 3 of the main text). This comparison shows that momentum relaxation is slower than decay of ballistic beam, which could be expected, based on that ℓ^{-1}_{mfp} is more sensitive to the large momentum transfer upon scattering, whereas ℓ^{-1} is caused by both large and small momentum transfers. Both of these two quantities are temperature-dependent, indicating the contribution of inelastic scattering processes, most likely, generated by phonons. In contrast to that, their difference, shown in Fig.S7 for the overlapping temperature interval for the available data using empty circles, is almost temperature-independent, suggesting that the low-angle scattering in the system is mostly elastic, rather than inelastic.

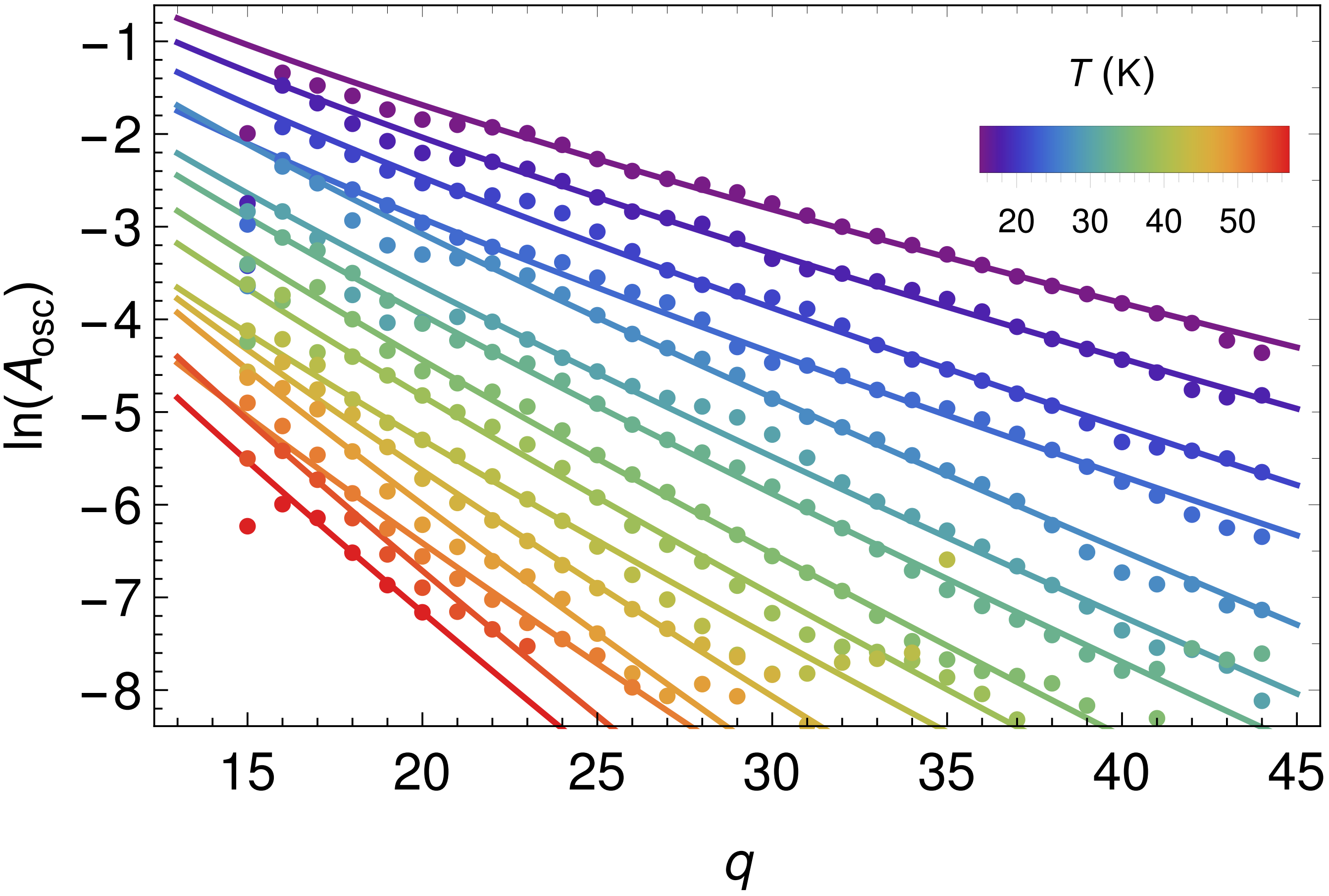

FIG. S6. Full data for $\ln A_{\mathrm{AB}}$ as a function of q for different temperatures, the lines correspond to $\ln A_{\mathrm{AB}} = a_0 - \frac{2\mathcal{L}}{\ell} - \ln(q)$ fit according to Eq.(4) of the main text (note that $\mathcal{L}(q) \approx \frac{\hbar}{eB}\, 2\,\mathrm{nm}^{-1} \propto q$, producing the main contribution to slope of the lines).

[1] P. Tomić, P. Rickhaus, A. Garcia-Ruiz, G. Zheng, E. Portolés, V. Fal'ko, K. Watanabe, T. Taniguchi, K. Ensslin, T. Ihn, and F. K. de Vries, Scattering between minivalleys in twisted double bilayer graphene, Phys. Rev. Lett. **128**, 057702 (2022).

[2] R. K. Kumar, X. Chen, G. Auton, A. Mishchenko, D. A. Bandurin, S. V. Morozov, Y. Cao, E. Khestanova, M. B. Shalom, A. Kretinin, *et al.*, High-temperature quantum oscillations caused by recurring Bloch states in graphene superlattices, Science **357**, 181 (2017).

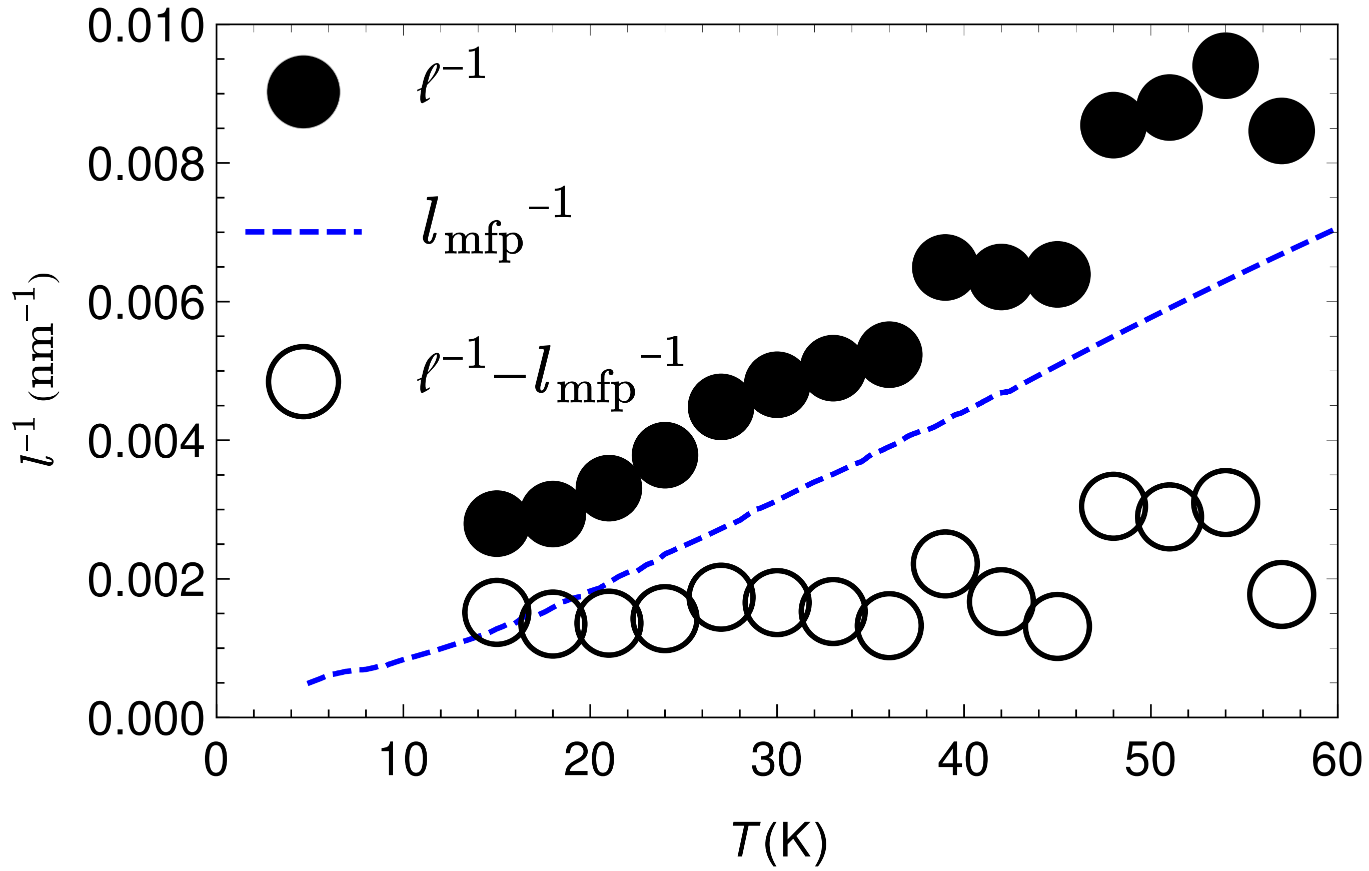

FIG. S7. Length ℓ vs the mean free path, $l_{\rm mfp}$ extracted from conductivity. We plot ℓ^{-1}, $l^{-1}_{\rm mfp}$ and the difference $\ell^{-1} - l^{-1}_{\rm mfp}$, showing weak temperature dependence of dephasing contributions.

[3] S. C. Creagh, J. M. Robbins, and R. G. Littlejohn, Geometrical properties of Maslov indices in the semiclassical trace formula for the density of states, Phys. Rev. A **42**, 1907 (1990).

[4] M. Wilkinson, Critical properties of electron eigenstates in incommensurate systems, Proc. R. Soc. Lond. A **391**, 305 (1984).

[5] L. C. Davis and S. H. Liu, Landau spectrum and line broadening in real metals, Phys. Rev. **158**, 689 (1967).

[6] A. Alexandradinata and L. Glazman, Semiclassical theory of Landau levels and magnetic breakdown in topological metals, Phys. Rev. B **97**, 144422 (2018).

[7] A. Alexandradinata, C. Wang, W. Duan, and L. Glazman, Revealing the topology of Fermi-surface wave functions from magnetic quantum oscillations, Phys. Rev. X **8**, 011027 (2018).

[8] L. M. Roth, Theory of bloch electrons in a magnetic field, Journal of Physics and Chemistry of Solids **23**, 433 (1962).

[9] A. Garcia-Ruiz, H.-Y. Deng, V. V. Enaldiev, and V. I. Fal'ko, Full Slonczewski-Weiss-McClure parametrization of few-layer twistronic graphene, Phys. Rev. B **104**, 085402 (2021).

[10] M. Lee, J. R. Wallbank, P. Gallagher, K. Watanabe, T. Taniguchi, V. I. Fal'ko, and D. Goldhaber-Gordon, Ballistic miniband conduction in a graphene superlattice, Science **353**, 1526 (2016), https://www.science.org/doi/pdf/10.1126/science.aaf1095.
